# Supplementary material for: Association between oral microbiome and seven types of cancers in East Asian population: a two-sample Mendelian randomization analysis
Source: Front Mol Biosci. 2023 Nov 21;10:1327893. doi: 10.3389/fmolb.2023.1327893 (PMC10702768; doi:10.3389/fmolb.2023.1327893)
Supplement: Supplementary file 1 [file DataSheet1.ZIP › Supplementary Materials/MR plots for tongue/tongue═╝/Colorectal cancer/pheno.2468_to_colorectal cancer_leave_one_out.pdf]

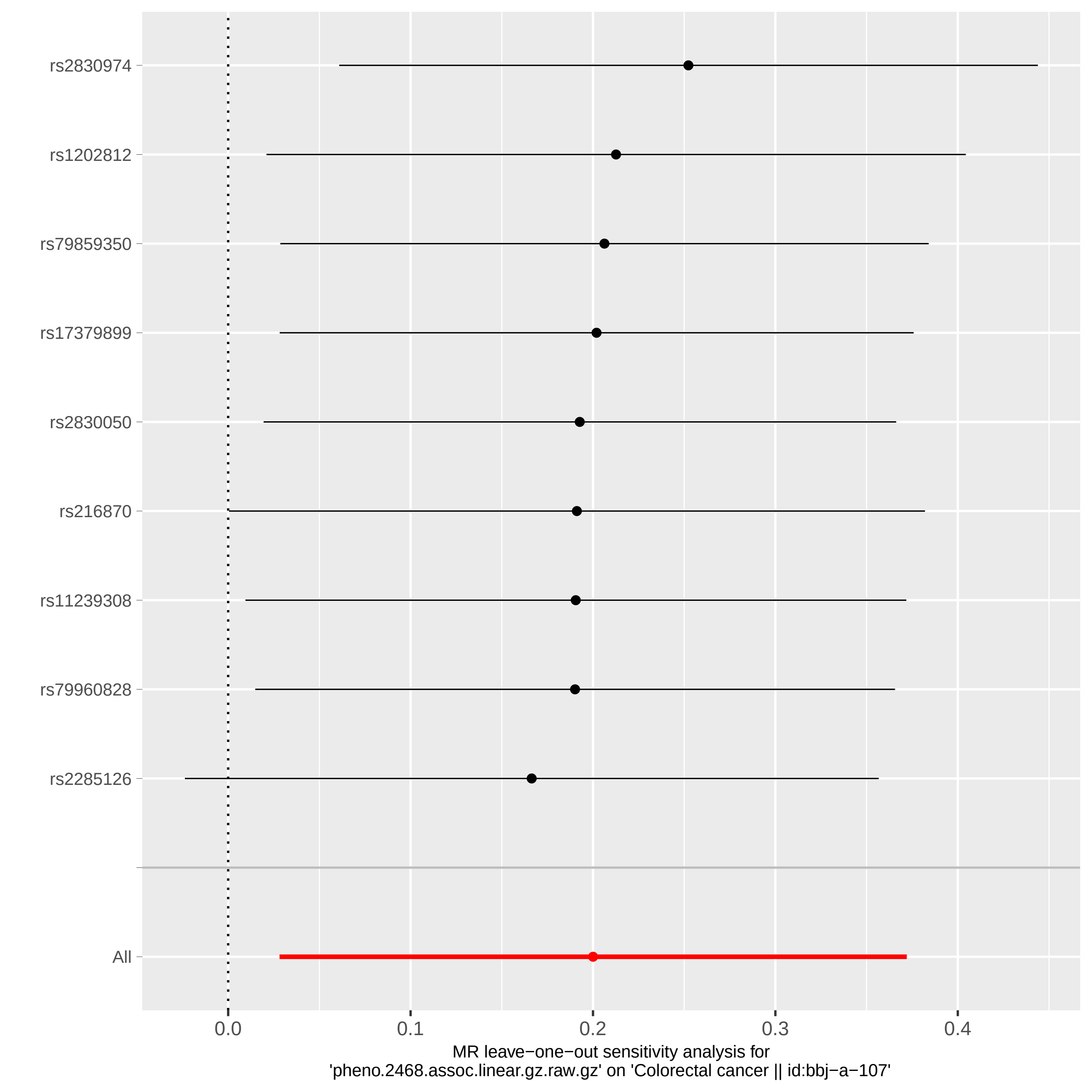
rs2830974
rs1202812
rs79859350
rs17379899
rs2830050
rs216870
rs11239308
rs79960828
rs2285126
All
0.0
0.1
0.2
0.3
0.4
MR leave−one−out sensitivity analysis for
'pheno.2468.assoc.linear.gz.raw.gz' on 'Colorectal cancer || id:bbj−a−107'
